# Transition from an in-person to a telemedicine diabetic retinopathy screening program

**DOI:** 10.5935/0004-2749.2023-0200

**Published:** 2024-03-27

**Authors:** Vanessa de O. Almeida Barbieri, Luis Filipe Nakayama, Gabriel Almeida Barbieri, Suzane Eberhart Ribeiro da Silva, Daniel Cunha José Karmouche, Marcelle Naomi Oshiro Shinzato, Rodrigo Eiji Nakagawa, Caio Vinicius Regatieri, Fernando Korn Malerbi

**Affiliations:** 1 Retina and Vitreous Sector, Hospital São Julião, Campo Grande, MS, Brazil; 2 Department of Ophthalmology, Universidade Federal de São Paulo, São Paulo, SP, Brazil; 3 Laboratory for Computational Physiology, Massachusetts Institute of Technology, Cambridge, MA, United States of America; 4 Universidade Federal de São Paulo, São Paulo, SP, Brazil

**Keywords:** Telemedicine/methods, Diabetic retinopathy, Diagnostic screening programs, Vision screening, Practice patterns, physicians

## Abstract

**Purpose:**

Timely screening and treatment are essential for preventing diabetic retinopathy blindness. Improving screening workflows can reduce waiting times for specialist evaluation and thus enhance patient outcomes. This study assessed different screening approaches in a Brazilian public healthcare setting.

**Methods:**

This retrospective study evaluated a telemedicine-based diabetic retinopathy screening implemented during the COVID-19 pandemic and compared it with in-person strategies. The evaluation was conducted from the perspective of a specialized referral center in an urban area of Central-West Brazil. In the telemedicine approach, a trained technician would capture retinal images by using a handheld camera. These images were sent to specialists for remote evaluation. Patient variables, including age, gender, duration of diabetes diagnosis, diabetes treatment, comorbidities, and waiting time, were analyzed and compared.

**Results:**

In total, 437 patients with diabetes mellitus were included in the study (mean age: 62.5 ± 11.0 years, female: 61.7%, mean diabetes duration: 15.3 ± 9.7 years, insulin users: 67.8%). In the in-person assessment group, the average waiting time between primary care referral and specialist evaluation was 292.3 ± 213.9 days, and the referral rate was 73.29%. In the telemedicine group, the average waiting time was 158.8 ± 192.4 days, and the referral rate was 29.38%. The telemedicine approach significantly reduced the waiting time (p<0.001) and significantly lowered the referral rate (p<0.001).

**Conclusion:**

The telemedicine approach significantly reduced the waiting time for specialist evaluation in a real-world setting. Employing portable retinal cameras may address the burden of diabetic retinopathy, especially in resource-limited settings.

## INTRODUCTION

The increase in the number of individuals with diabetes mellitus is challenging for health systems globally. As of 2021, more than 500 million people worldwide are affected by diabetes, and the number is expected to rise to 784 million by 2045^(1)^. Diabetic retinopathy (DR) is among the most common complications of diabetes. DR affects nearly one-third of diabetic patients and may cause blindness^(2)^. To prevent such a severe outcome, the International Council of Ophthalmology and the American Diabetes Society recommend periodic retinal examinations^(3^,^4)^. By promoting regular screenings, early diagnosis of DR and prompt intervention for preserving patients’ vision and quality of life become possible^(5)^.

With Brazil’s population being 211 million, it is a country of continental dimensions. Approximately 80% of its residents depend exclusively on the public health-care system for their medical needs^(6)^. The country’s healthcare system is systematized into primary, secondary, and tertiary care, with the primary care physician being the initial point of contact for patients. For general ophthalmological screening, patients are referred to specialized care. Less complex issues in primary care are well managed by adopting a tiered approach to healthcare delivery, whereas more complex cases are referred to secondary and tertiary care.

Going from primary care to specialist care takes a long time, and several reasons contribute to these long waiting periods, including the shortage of specialized professionals, lack of referral and counter-referral protocols and criteria, inadequate queue priority organization, and barriers to access. This delay is a significant risk for patients with irreversible blindness-causing DR who require timely treatment. Currently, a widespread DR screening strategy is lacking in Brazil^(6)^. Consequently, several regional initiatives have been taken for mass screening of DR^(7)^.

The COVID-19 pandemic resulted in city-wide lock-downs and suspension of ambulatory healthcare services, instigating the rapid implementation of digital and remote healthcare solutions, including telemedicine. These innovative approaches have offered significant benefits, particularly to the ophthalmology field. Imaging techniques, including retinal fundus photographs and optical coherence tomography, have allowed the screening of ophthalmological diseases, including DR^(8)^. The implementation of telemedicine in ophthalmology can potentially revolutionize the field as more efficient and accessible care can be offered to patients^(9^,^10)^. Moreover, telemedicine has already been proven to be cost-effective for DR screening^(10)^.

This study compared two screening approaches for DR, namely the traditional, in-person strategy and a telemedicine-based approach, within an urban, public healthcare referral center in Brazil. It also evaluated whether a telemedicine approach can reduce the waiting time for specialist evaluation.

## METHODS

This retrospective study included patient data from 2019 to 2022 and was conducted at São Julião Hospital, Campo Grande, Mato Grosso do Sul, Central-West Brazil. This hospital encompasses 131 beds and has a team of 38 ophthalmologists and 27 nurses. This study was conducted in accordance with the Helsinki principles and was approved by the local IRB.

Data were collected from diabetic patients who underwent retinal examinations during the study period, following two opportunistic strategies. The transition of strategies coincided with the COVID-19 pandemic. The analysis was conducted from the perspective of a referral center, where patients were first examined in person and then subjected to telemedicine evaluation. In-person examination was conducted only for patients who developed referable retinal disease after the transition to telemedicine due to the COVID-19 pandemic.

### Screening strategies

The first strategy was named the “in-person screening” strategy. In this approach, a trained ophthalmologist performed binocular indirect ophthalmoscopy in patients referred by a primary care physician. This procedure was performed in a specialized care setting, after pharmacological mydriasis (tropicamide 1%, one drop every 5 min, two times). This strategy was implemented from 2019 to 2021.

**Figure 1:**
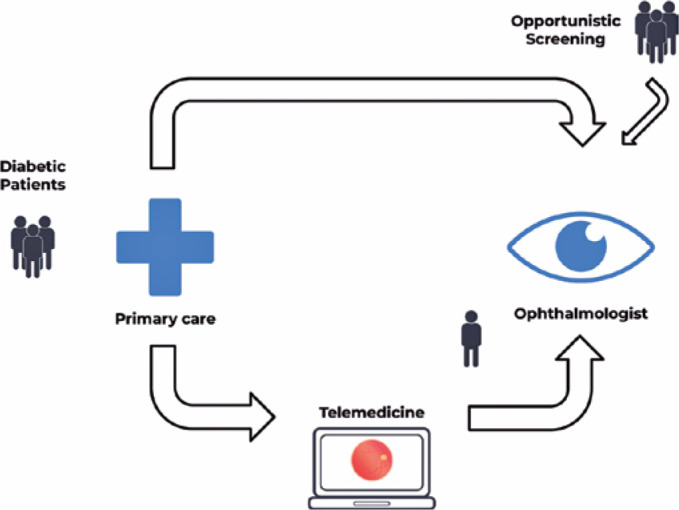
Flowchart of diabetic patients. The in-person screening process at the upper part and the Telemedicine program at the lower part.

The second strategy was termed the “telemedicine strategy.” In this approach, a standardized retinal imaging protocol was implemented in patients referred by a primary care physician, followed by remote expert interpretation of images in a store-and-forward manner^(11)^ (Figure 1).

Trained technicians captured all the retinal fundus photographs by using the handheld portable Phelcom Eyer retinal camera (Phelcom Technologies, São Carlos, Brazil).

The Eyer, developed by Phelcom Technologies (Phelcom Technologies, LLC, Massachusetts, USA), uses a Samsung Galaxy S10 smartphone (running Android 11) as its foundation. This handheld cellphone camera with a 45° field angle and 12-megapixel sensor is designed to capture retinal fundus photographs. These features result in images of 1600 × 1600 pixels. Notably, the Eyer has an autofocus capability spanning from -20 to +20 diopters. This strategy was implemented from 2021 to 2022.

### Labeling protocol

Diabetic retinal lesions, which include hemorrhages, microaneurysms, venous beading, intraretinal microvascular abnormalities, new vessels, vitreous or preretinal hemorrhage, and the presence of retinal tractional membranes, were evaluated in accordance with the guidelines of the International Classification of Diabetic Retinopathy (ICDR). DR severity was classified as follows: no DR, mild nonproliferative DR, moderate nonproliferative DR, severe nonproliferative DR, proliferative DR, or ungradable. Patients with more than moderate DR in any eye were categorized as referable. Based on the criteria established by the ICDR, the presence of diabetic macular edema (DME) was determined by identifying retinal thickening covering at least one disk area from the central fovea. Patients detected with pan-retinal photocoagulation scars on images were considered to have proliferative DR.

### Comparative analysis

For comparing both strategies, we evaluated the clinical and demographic variables of patients from each group: patients’ age, gender, diabetes diagnosis time, use of insulin, presence of systemic arterial hypertension, DR classification, and the percentage of patients with DME. The waiting time between primary care and ophthalmological examination was also compared between both strategies.

The preand post-COVID-19 data, starting from March 2020, were compared to assess the influence of the pandemic on waiting time.

### Statistical analysis

We compared patient waiting time and their clinical and demographic variables between the in-person and telemedicine groups. A chi-square test was conducted to compare categorical variables, and a Mann-Whitney test was conducted to compare continuous variables. A 0.05 significance level was used to determine statistical significance. The statistical analysis was conducted and plots were drawn using Python 3.9 and Python libraries (seaborn and matplotlib).

## RESULTS

In total, 437 patients (265 women (60.64%)) were evaluated at the referral center during the study period. The mean patient age was 62.46 years (median: 64, standard deviation (SD): 11.02). The mean diabetes duration was 15.32 years (median: 15, SD: 9.72), and the percentage of insulin users was 67.85%. Systemic arterial hypertension was noted in 77.73% of the patients. Table 1 presents clinical and demographic information, and differences in patient characteristics among both strategies.

**Table 1:** Comparative demographics and clinical characteristics between the groups

	Total	In-person	Telemedicine	χ^^2^^	p
**Gender, female, n (%)**	**265 (60.64)**	**166 (65.39)**	**99 (61.75)**	**161**	**0.688**
**Age, y (SD)**	**62.46 (11.02)**	**61.41 (10.45)**	**64.30 (11.74)**		0.007
**Waiting time, d (SD)**	**243.40 (192.44)**	**292.27 (213.94)**	**158.81 (192.44)**		<0.001
**Diabetes time, y (SD)**	**15.32 (9.72)**	**17.28 (9.745)**	**11.99 (8.73)**		<0.001
**Insulin**	**287 (77.73)**	**165 (62.26)**	**122 (77.22)**	10.143	0.001
**Systemic arterial hypertension**	**328 (77.73)**	**200 (75.76)**	**128 (81.01)**	1.577	0.209
**Referral**	**250 (57.21)**	**203 (73.29)**	**47 (29.38)**	79.879	<0.001

**Figure 2:**
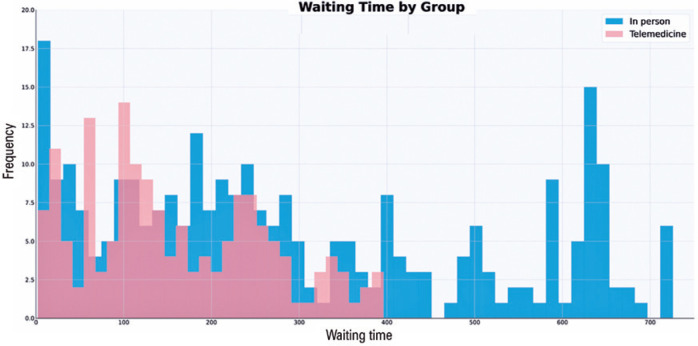
Histogram of the waiting times between the in-person and telemedicine programs.

The in-person group had 277 patients with a mean age of 61.40 years (SD: 10.45). Of these patients, 58.47% were female patients. The mean diabetes duration and the percentage of insulin users were 17.28 years (SD 9.75) and 62.26%, respectively, in the in-person group. The time period from primary care referral to the ophthalmologist assessment was 292.27 days (median: 235, SD: 213.94), with 203 referred patients (Figure 2).

The telemedicine group included 160 patients with a mean age of 64.30 years (SD: 11.74). Of these patients, 61.66% were female patients. The mean diabetes duration and percentage of insulin users were 11.99 years (SD: 8.74) and 77.21%, respectively, in the telemedicine group. The time period from primary care referral to the ophthalmologist assessment was 158.81 days (median: 143, SD: 103.65), with 47 referred patients (Figure 2). In the telemedicine group, of 1,748 retinal images, 41 images (2.34%) were ungradable. Patients with these ungradable images were also referred for specialist evaluation.

Statistically significant differences in waiting times (p<0.001), diabetes duration (p<0.001), insulin use (p=0.001), and referral rate (p<0.001) were observed between the groups (Table 1).

In the COVID-19 influence analysis, the average waiting time before COVID-19 was 655 days (median: 644, SD: 33), whereas that after COVID-19 was 208 days (median: 183, SD: 155.19).

## DISCUSSION

According to our study, the telemedicine approach significantly reduced the waiting time from primary care referral to specialist evaluation compared with the in-person approach. Studies have demonstrated that telemedicine can improve healthcare coverage and reduce waiting time for medical evaluation^(8^,^11^,^12)^. The telemedicine screening approach was more efficient than the conventional referral model and opportunistic screening methods.

The traditional, in-person approach, which was the standard before the COVID-19 pandemic at the hospital, is clearly impractical and unsustainable because the global diabetic population is rapidly increasing. By the year 2030, every ophthalmologist may have to contribute over 4.5 million hours per year to evaluate all diabetic patients at least once annually^(8)^. Modern, technology--enriched approaches such as telemedicine and artificial intelligence (AI) are required to bridge this enormous gap and allow evidence-based eye care to reach a considerably broader population^(8)^.

In the telemedicine approach, specialists remotely evaluate images and reserve in-person evaluations for individuals with sight-threatening diseases. This approach is a significantly more sustainable alternative than the traditional approach. This approach has already been evaluated in our setting, demonstrating high agreement between in-person consultations and telemedicine assessments. This assessment was conducted in both a multicenter study and a real-world, high-burden setting^(13)^. This telemedicine strategy offers an optimized workflow, thereby allowing experts to dedicate more time to treating patients with severe diseases rather than examining those without severe retinal conditions. Telemedicine consequently led to a significant reduction in waiting times for specialist evaluations. Moreover, the comparison between the groups revealed that patients from the telemedicine group had less severe DR and a lower referral rate. This remarkable finding may be directly related to the difference in waiting times because DR progresses over time, and timely referral is a milestone for preventing blindness.

Implementing telemedicine screening programs in low and middle-income countries (LMICs) can be challenging because of several factors, including the high cost of equipment, limited internet connectivity, and outdated pricing standards. Despite these challenges, portable cameras that can connect to smartphones have facilitated the adoption of telemedicine in these LMICs. These devices are affordable, easy to use, and provide high-quality images, as observed by the low ungradable rate reported herein (2.34%). Such portable cameras also allow efficient remote diagnosis and monitoring of various medical conditions other than DR. These cameras may transform healthcare delivery in LMICs, where access to specialized medical care is limited^(11^,^13^,^14)^.

Although no automated steps were employed during screening, AI-enabled systems allow improvement in the efficiency of DR screening^(15^,^16)^. Automated screening for image quality and DR detection can allow further enhancement of the screening process and streamlined workflow. Future studies could explore the feasibility and effectiveness of integrating AI-enabled systems into telemedicine screening programs for DR to improve patient outcomes and resource utilization.

The present study mainly contributes to the idea of real-world implementation of a telemedicine program for DR screening in response to the COVID-19 pandemic. Significantly shorter waiting times and lower referral rates were observed following the adoption of this innovative approach in a public healthcare setting in Brazil. However, a telemedicine approach does not entirely substitute an in-person, specialist evaluation. Telemedicine enables efficient and convenient screening for DR; however, it does limit the scope of the ophthalmological examination to imaging analysis. Optimizing the screening process could provide more equitable access to specialized healthcare because constraints on the availability of a specialized workforce can be experienced worldwide.

The pandemic influence analysis demonstrated lower waiting times despite COVID-19. However, the limited number of cases, the reduction in ambulatory patients, and the beginning of the telemedicine system contributed to this result.

While our study highlights the advantages of telemedicine for DR screening, its limitations must also be acknowledged. Although different strategies during a transition were compared, the study groups were not homogenous. Such heterogeneity, which was partly influenced by the COVID-19 lockdown and reduction in ambulatory care during that period, may have limited our conclusions and introduced bias in the analysis. As an example, the traditional strategy group evidently included patients with more severe diseases, as can be concluded from the longer diabetes duration and higher referral rates. Additionally, our study did not evaluate the cost-effectiveness of implementing telemedical screening programs. Further economic studies are warranted to assess the feasibility of deploying such programs in LMICs. Once these limitations are addressed in future studies, a more comprehensive understanding of telemedicine’s potential benefits and challenges in DR screening can be attained. Several challenges related to preventable blindness remain, as evidenced by the still--long waiting period reported for specialist evaluation even with the use of the telemedicine approach. These challenges also need to be addressed in future studies.

Our study highlights the successful implementation of a low-cost retinal camera and a telemedicine screening program for DR in a real-world public referral center. This strategy leverages the expertise of healthcare professionals in capturing retinal images and remote ophthalmologists in conducting the screening process, thereby effectively addressing the COVID-19 pandemic--presented challenges. The strategy provided a solution for continuity of care during these unprecedented times as well as facilitated an optimized workflow that likely enhanced patient access to healthcare services. Our study findings suggest that telemedicine screening programs involving portable retinal cameras and remote ophthalmologists have the potential to address the burden of DR, particularly in resource-limited settings.

AUTHORS’ CONTRIBUTION

**Substantial contribution to conception and design:** Vanessa de O. Almeida Barbieri, Luis Filipe Nakayama, Gabriel Barbieri, Suzane Eberhart Ribeiro da Silva, Daniel Cunha José Karmouche, Marcelle Naomi Oshiro Shizato, Rodrigo Eiji Nakagawa, Caio Vinicius Regatieri and Fernando Korn Malerbi. **Acquisition of data:** Gabriel Barbieri, Suzane Eberhart Ribeiro da Silva, Daniel Cunha José Karmouche, Marcelle Naomi Oshiro Shizato and Rodrigo Eiji Nakagawa. **Analysis and interpretation of data:** Vanessa de O. Almeida Barbieri, Luis Filipe Nakayama, Gabriel Barbieri, Suzane Eberhart Ribeiro da Silva, Daniel Cunha José Karmouche, Marcelle Naomi Oshiro Shizato, Rodrigo Eiji Nakagawa, Caio Vinicius Regatieri and Fernando Korn Malerbi. **Drafting of the manuscript:** Vanessa de O. Almeida Barbieri, Luis Filipe Nakayama, Gabriel Barbieri, Suzane Eberhart Ribeiro da Silva, Daniel Cunha José Karmouche, Marcelle Naomi Oshiro Shizato, Rodrigo Eiji Nakagawa, Caio Vinicius Regatieri and Fernando Korn Malerbi. **Critical revision of the manuscript for important intellectual content:** Vanessa de O. Almeida Barbieri, Luis Filipe Nakayama, Gabriel Barbieri, Suzane Eberhart Ribeiro da Silva, Daniel Cunha José Karmouche, Marcelle Naomi Oshiro Shizato, Rodrigo Eiji Nakagawa, Caio Vinicius Regatieri and Fernando Korn Malerbi. **Have given final approval of the submitted manuscript:** Vanessa de O. Almeida Barbieri, Luis Filipe Nakayama, Gabriel Barbieri, Suzane Eberhart Ribeiro da Silva, Daniel Cunha José Karmouche, Marcelle Naomi Oshiro Shizato, Rodrigo Eiji Nakagawa, Caio Vinicius Regatieri and Fernando Korn Malerbi. **Statistical analysis:** Luis Filipe Nakayama. **Obtaining funding:** None. **Administrative, technical, or material support supervision:** None. **Research group leadership:** Caio Vinicius Regatieri and Fernando Korn Malerbi.
